# Point OutWords: protocol for a feasibility randomised controlled trial of a motor skills intervention to promote communicative development in non-verbal children with autism

**DOI:** 10.1186/s13063-019-3931-1

**Published:** 2020-01-23

**Authors:** Ailbhe McKinney, Kathryn L. Hotson, Alicia Rybicki, Emma J. L. Weisblatt, Claudia Días, Juliet Foster, Sofía S. Villar, Suzanne Murphy, Matthew K. Belmonte

**Affiliations:** 1 Division of Psychology, Nottingham Trent University, Nottingham, UK; 2 School of Psychology, University of Birmingham, Birmingham, UK; 3 Department of Psychology, University of Cambridge, Cambridge, UK; 4 Peterborough Integrated Neurodevelopmental Service, Cambridgeshire and Peterborough NHS Foundation Trust, Peterborough, UK; 5 Institute of Psychiatry, Psychology and Neuroscience, King’s College London, London, UK; 6 MRC Biostatistics Unit, School of Clinical Medicine, University of Cambridge, Cambridge, UK; 7 Institute for Health Research, University of Bedfordshire, Bedford, UK; 8 The Com DEALL Trust, Bangalore, India

**Keywords:** Autism spectrum disorder, Minimally verbal, Non-verbal, Motor, Language, Communication, iPad, Feasibility, Randomised controlled trial

## Abstract

**Background:**

*Point OutWords* is a caregiver-delivered, iPad-assisted intervention for non-verbal or minimally verbal children with autism. It aims to develop prerequisite skills for communication such as manual and oral motor skills, sequencing, and symbolic representation. This feasibility trial aims to determine the viability of evaluating the clinical efficacy of Point OutWords.

**Methodology:**

We aim to recruit 46 non-verbal or minimally verbal children with autism and their families, approximately 23 per arm. Children in the intervention group will use Point OutWords for half an hour, five times a week, for 8 weeks. Children in the control group will have equal caregiver-led contact time with the iPad using a selection of control apps (e.g. sensory apps, drawing apps). Communication, motor, and daily living skills are assessed at baseline and post-intervention. Parents will keep diaries during the intervention period and will take part in focus groups when the intervention is completed.

**Discussion:**

Point OutWords was developed in collaboration with children with autism and their caregivers, to provide an intervention for a subgroup of autism that has been historically underserved. As autism is a heterogeneous condition, it is unlikely that one style of intervention will address all aspects of its symptomatology; the motor skills approach of Point OutWords can complement other therapies that address core autistic symptoms of social cognition and communication more directly. The current feasibility trial can inform the selection of outcome measures and design for future full-scale randomised controlled trials of Point OutWords and of other early interventions in autism.

**Trial registration:**

ISRCTN, ISRCTN12808402. Prospectively registered on 12 March 2019.

## Background

Designing and testing interventions targeting communication skills in children with autism has proven difficult given its heterogeneous presentation and the lack of measures sensitive to change over time [1], yet substantive progress has taken place in recent years [2–4]. Applied behaviour analysis (ABA) [5] is effective but not routinely provided in most jurisdictions as its labour-intensive nature presents an economic barrier. The UK National Institute for Health and Care Excellence (NICE) guidelines [6] recommend a combination of social skills groups, occupational therapy and speech/communication therapy, with most children receiving just 6–8 sessions. The most successful treatment studies have targeted children’s socially engaged imitation [7] or parents’ evocation of children’s communicative behaviours, producing long-term social communicative improvements [8, 9] but often at significant economic cost [10] and without effectiveness for all children. The Early Start Denver Model intervention combines principles of ABA with adult responsivity and sensitivity to child cues, and has demonstrated effectiveness [11], although the only domain of improvement replicated across studies is language, and not developmental quotient, autism severity, or adaptive behaviour [12]. Alternative or complementary approaches to autism therapy are the several unproven, uncontrolled and in many circumstances invalid parent-delivered methods of manual motor symbolic communication [13, 14]. These demand extensive training for those who implement them, are labour-intensive, and can be difficult to subject to controlled testing [15]. Our novel intervention trains manual motor skills and symbolic representation and is facilitated by our freely downloadable iPad app, Point OutWords™ [16], yielding light caregiver training - just 30 min by a research assistant with a 4-page instruction booklet. Built into Point OutWords are automated measures of motor performance, and of any caregiver-initiated movements of the device that might cue particular responses.

Our research and that of others has demonstrated strong association between language delay and motor dysfunction in a significant subgroup of autism [17–23]. In our own clinical sample, fully one third of autistic children who lack communicative speech manifest a distinctive pattern in which motor and particularly oral motor skills are impaired whilst expressive language is impaired disproportionately to more intact receptive language [19] - the inverse of the more general pattern of receptive impairment in autism [24]. Our work suggests that the high-level deficits in social communication so overtly characteristic of autism may emerge developmentally from interaction of lower-level, more subtle, more fundamental traits in three general areas: social motivation and reward, cognitive and motor control, and sensory perception [25], and thus implies that early training directed towards such lower-level skills - in the case of this new intervention, motor control and sequencing - might exert a positive knock-on effect on the development of communicative skills. This approach aims to develop social communicative skills by training non-social, domain-general prerequisite skills [26] - implementing what is in a sense a “back door” route to autism therapy.

Children with autism who are non-verbal or minimally verbal are often excluded from research [27], and children with severe communication impairments are less likely to be included in treatment studies [28]. Point OutWords was developed with the inclusion of clients with autism both explicitly by asking children to choose amongst graphical design options and by obtaining therapists’ feedback on their behalf, and implicitly by observing children’s preferences. Point OutWords was designed with the strengths of autistic cognition in mind. Many children with autism excel at detail-oriented tasks such as jigsaws due to their abnormally strong ability to perceive local details. Whereas many teaching and learning strategies adapted from methods for non-autistic people end up working *against* autistic cognition by asking people with autism to do what they cannot easily do, Point OutWords works *with* autistic cognition, by beginning from this point of strength [29]. Indeed, our pilot study (*N* = 7) [30] indicated that children and parents enjoyed using Point OutWords.

The current trial will evaluate the feasibility of testing the Point OutWords intervention, focusing on usability and engagement, fidelity to treatment, and recruitment and retention for a future full-scale randomised controlled trial (RCT). It will assess variance in test-retest measures, informing the selection of suitable outcome measures sensitive to change and determining the target sample size of a large-scale randomised controlled trial, key steps in designing RCTs for autism interventions [3, 29].

### Aims

Our primary aim is to determine whether the clinical efficacy of Point OutWords as an intervention for enhancing communication and interaction in minimally verbal or non-verbal children with autism can be feasibly evaluated. Feasibility will be assessed based on the outcomes as follows:

1. Intervention fidelity and acceptability - Establishing whether Point OutWords can be delivered by parents based on frequency, duration, and content of intervention sessions, in addition to parents’ understanding of the intervention and proficiency in delivering it; assaying whether the intervention is accepted by children and how the intervention fits into the family routine.
2. Recruitment and retention - establishing whether sufficient participants can complete a future full-scale trial based on participation rates, drop-out, and completion rates; determining whether sufficient participants match inclusion criteria, including impairment in motor skills and expressive language.
3. Outcome measure efficacy - assessing whether candidate measures of motor and communicative function, alongside measures of individual and family social function, are acceptable and sensitive test-retest measures in the target population; ascertaining variance in outcome measures will inform the sample size of a future RCT.

Positive outcomes from this feasibility study would lead to a subsequent full-scale RCT. Point OutWords carries potential for substantial economic benefit in the UK National Health Service (NHS) and other settings because it is designed for use by caregivers with minimal training.

## Methodology

### Design

**Fig. 1:**
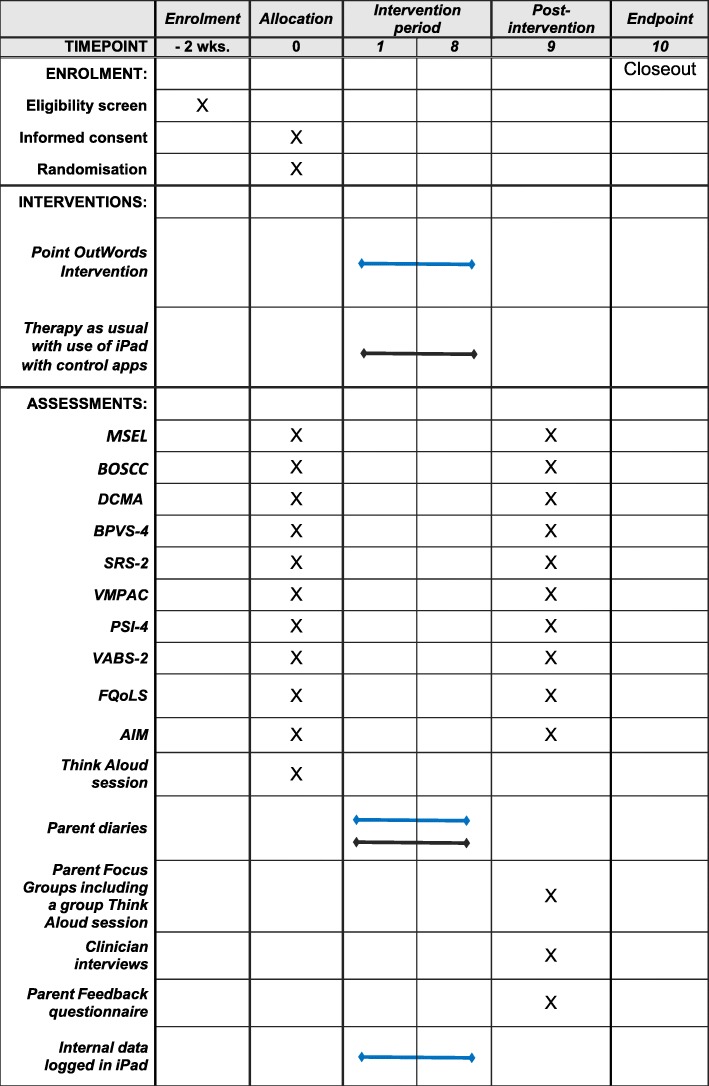
Standard Protocol Items: Recommendations for Interventional Trials (SPIRIT) figure with timeline for recruitment, assessments, and interventions. Schedule of assessments: *MSEL* Mullen Scales of Early Learning, *BOSCC* Brief Observation of Social Communication Change, *DCMA* Dyadic Communication Measure for Autism, *BPVS-3* British Picture Vocabulary Scale, *SRS-2* Social Responsiveness Scale, *VMPAC* Verbal Motor Production Assessment for Children, *PSI-4* Parenting Stress Index, *VABS-2* Vineland Adaptive Behavior Scales, *FQoLS* Family Quality of Life Scale, *AIM* Autism Impact Measure. Blue lines indicate the intervention group and black lines indicate the control group

The study applies a feasibility, parallel-groups, randomised controlled design, in which participants are randomly allocated to the intervention group or the control group at a 1:1 ratio. Randomisation will be stratified by gender given the uneven gender ratio in autism diagnoses. The intervention period will last 8 weeks. Figure 1 shows assessments to be administered at baseline and immediately post-intervention. This trial protocol is in line with Standard Protocol Items: Recommendations for Interventional Trials (SPIRIT) 2013 (see SPIRIT Checklist in Additional file 1).

### Study setting

The intervention will take place within the homes of participants in the Peterborough area. Psychometric testing will be administered at the family home, the child’s school, or at the Cambridgeshire and Peterborough NHS Foundation Trust (CPFT) Integrated Children’s Service based in the City Care Centre, Peterborough, UK, depending on the preference of the children and their families.

### Participants

#### Recruitment

Participants will be recruited through CPFT Integrated Children’s Services and through community paediatric services in nearby NHS trusts. Parents or caregivers will be approached by known and trusted clinicians, a strategy shown to heighten recruitment rates [31]. Parents will receive a recruitment invitation letter followed by an information sheet for the study. In addition to talking to their child’s consultant, parents will have the opportunity to talk to a research assistant over the phone or via email to ask questions.

#### Inclusion criteria

The inclusion criteria are:

- Ages 3–15 years.
- Autism diagnosis confirmed by multidisciplinary assessment as part of routine NHS clinical care, and meeting criteria for autism on the Autism Diagnostic Observation Schedule-Generic (ADOS-G) [32].
- Nonverbal or minimally verbal (lacking communicative speech or speaking fewer than 100 single words and no phrase speech).
- Clinical judgement of motor impairment.
- Clinical judgement that age-adjusted receptive language surpasses age-adjusted expressive language.
- English is the primary language spoken in the home (as Point OutWords is not yet translated).

#### Exclusion criteria

The exclusion criteria are:

- Severe visual or hearing impairment (which would affect interaction with an iPad).
- Caregiver does not speak English and therefore would not be able to administer the intervention.
- Severe impairment of motor function (e.g. cerebral palsy).

### Sample size

We aim to recruit 46 families (approximately 23 per arm). Another RCT of a similar population [9] recruited 62.8% of (152 of 242) families assessed for eligibility. A target of 46 families would let us estimate a recruitment rate of 62.8% within a 95% confidence interval of ±14%. In the unlikely event of falling far short of that norm, then we would recommend modifications to the recruitment strategy.

### Allocation and blinding

Individuals will be randomly allocated to the intervention or control group. Participants will be block-randomised, stratified by gender, by the web-based randomisation service Sealed Envelope™ [33], overseen by a trial statistician uninvolved in data collection. Upon obtaining written consent, a research assistant will access Sealed Envelope™ to obtain the treatment allocation, and will therefore be aware of each participant’s allocation, but will be blind to the time point of psychometric assessments at the data analysis stage. Due to the nature of the intervention participants cannot assuredly be blinded to treatment, as they will be aware of the app they are using. The rest of the research team, including the principal investigator and clinical lead, will be blinded to the allocation of each family.

### Intervention

Point OutWords [30] is an iPad game developed to train fine motor and cognitive skills in children with autism. Participants in the experimental group will receive the Point OutWords intervention, with a target of at least 20 h of contact time, as an adjunct to standard NHS clinical therapy. The intervention will be administered by parents/caregivers in the home, either on a loaned iPad or on the family’s own iPad if preferred. Parents will receive training from the research team to use Point OutWords, and written instructions.

Point OutWords has three modes of increasing difficulty, which tap skills prerequisite to language and communication. Learning starts in the Point mode where the child uses their finger to drag jigsaw pieces to complete a picture. The object is segmented into jigsaw puzzle pieces, which are scattered round the touchscreen. Puzzles are images from daily life e.g. bath, shampoo. The child has the opportunity to develop the manual motor skills prerequisite to pointing to interact with a touchscreen. The touchscreen software interface is adapted so as to accommodate the autistic user’s open-loop style of visuomotor control and is customisable to meet the child’s motor ability.

From here, the child progresses to Type mode. In Type mode, puzzle pieces cannot be dragged into place in an iconic style of reference, but instead must be cued by symbolic reference: Each puzzle piece is labelled with a letter in the word, there are as many puzzle pieces as there are letters in the word, and in order to cause a piece to snap into place within the puzzle, the participant must press the corresponding letter key on a virtual keyboard that appears on the touchscreen. The Type mode aims to encourage symbolic representation, with which non-verbal children with autism show difficulty [34] and which underpins communication development [35]. In addition to improving motor skills, Point OutWords teaches sequencing through piecing several parts of a puzzle together. Cognitive sequencing underlies language development along with planning for social interaction and daily activities [26].

Finally, in the Speak mode the child assembles each jigsaw piece by speaking each syllable of a word in order, allowing the child to practise their oral motor skills. The object is segmented into a number of puzzle pieces equal to the number of syllables in the word. Each syllable is modelled, the client is prompted to speak each one, and OpenEARS speech recognition software is used to detect these pronunciations. Speaking a syllable causes the corresponding puzzle piece to snap into place. Tolerance for articulation errors and other slight inaccuracies of the match between actual and modelled pronunciations can be configured by the caregiver. Advancing to Speak mode will be difficult for the majority of children in this non-verbal/minimally verbal sample; we expect that most children will stop with Type mode.

Point OutWords is intended to be used with a parent or other caregiver who can support the child in an “errorless learning” format, filling in responses and correcting errors when the child is unable to do so independently, and redirecting the child towards the task when attention wanders. Progressing to a more difficult level is at the discretion of the caregiver in interaction with the child.

### Control group

Participants not receiving the experimental intervention receive standard NHS clinical therapy and equal iPad contact time using a selection of iPad apps, which include Magic Fluid Lite [36], Heat Pad - Relaxing Heat Sensitive Surface! [37], Draw with Stars! Play With Shooting Stars [38], Baby Drum [39], Doodle Buddy Paint Draw App [40], Aquarium Live HD [41], and Dogs [42]. Control apps were chosen based on their absence of skill promotion, particularly in areas Point OutWords aims to enhance, such as sequencing, assembling puzzles, vocabulary learning, fine motor skills training, social learning, or skills that underpin language development (e.g. category learning). The control group will receive equal regular support during the study period, and will be offered Point OutWords intervention after the end of the study.

### Adherence monitoring

Participants will have fortnightly contact with the research team via telephone calls to monitor fidelity to assigned intervention, and address queries related to the intervention or using the app(s). During telephone calls, consent for continuing participation is taken or, on the participant’s request, the intervention is stopped. iPad data logged during sessions will be used to monitor session frequency, session length, and games played. Participants will be asked to keep a diary during the intervention period, which includes questions related to frequency of iPad sessions, when during the day iPad sessions take place, and the types of games played. Phone calls and diaries will also record what other interventions, if any, the child or parents are taking part in.

### Outcomes

#### Primary outcomes

##### Child observations

**Mullen Scales of Early Learning (MSEL)**

The MSEL [43] is widely used as a measure of cognitive ability, motor ability, and language skills in research and clinical evaluations of children with autism [44, 45]. Relevant to the study, the fine motor, receptive language, and expressive language subscales will be administered. Each subscale yields developmental age equivalents, and therefore is applicable to developmentally delayed children outside the 68-month normed age range [46]. The MSEL shows good test-retest and inter-rater reliability [43] and strong concurrent validity with other developmental tests of motor and language, including the Vineland Adaptive Behaviour Scales II (VABS-II) [44]. Differences in mean scores from baseline to post-intervention between the intervention and control group will measure treatment efficacy. Separately, baseline mean scores will be used to characterise for whom the treatment is (or might be) effective. For example, a disparity in age-equivalent scores between the receptive and expressive language domain might predict treatment effects.

**Brief Observation of Social Communication Change (BOSCC)**

The BOSCC [47] is scored from a 12-min video recording of a parent-child play session, coded on 16 items assessing atypicality, frequency, and severity of autistic symptoms. The BOSCC coding scheme was developed from the Autism Diagnostic Observation Schedule (ADOS)-2 [48] by the same research team, to detect clinically significant changes in autistic symptoms following treatment. The BOSCC has yielded excellent inter-rater reliability and high test-retest reliability [47]. Treatment efficacy will be measured by differences in mean scores from baseline to post-intervention between the intervention and control groups.

**Dyadic Communication Measure for Autism (DCMA)**

The DCMA [8] is coded from the same parent-child play session as the BOSCC, to assess dyadic communicative interaction between parent and child, making it largely complementary to the behaviours scored by the BOSCC. Independent codes are applied to instances of the parent’s synchronous responses with the child, the child’s initiations with the parent, and the time spent in shared attention. The mean change from baseline to post-intervention will be compared between the intervention and control groups.

**British Picture Vocabulary Scale III (BPVS-III)**

The BPVS [49] provides a rapid measure of receptive vocabulary, tested via the same method - nonverbal pointing - that Point OutWords applies in its Point Mode (and builds on in Type Mode). Inclusion of this measure thus aims to assess both vocabulary itself and the ability to express vocabulary through pointing. The mean change from baseline to post-intervention will be compared between the intervention and control groups.

##### Parent reports

**Social Responsiveness Scale II (SRS-2)**

The SRS [50] assesses social communicative competence from 2.5 years onwards. Comprising 65 items, the SRS-2 is sensitive to behavioural change and widely used as an autism treatment outcome measure [51, 52]. Mean scores taken at baseline and post-intervention will be compared between the intervention and the control groups.

##### Feasibility and acceptability measures

**Internal iPad data**

Pseudonymised activity logs will be uploaded from the iPad. A time series of all stimuli and responses, number of completed puzzles, and total and average session length will be logged.

**‘Think-aloud’ sessions**

An optional session in which parents are recorded voicing their thoughts and experiences whilst using Point OutWords, the individual “think-aloud” session will be completed before the intervention period begins.

**Focus groups**

An optional group interview will be held after participation in the study, for the intervention and control groups separately. Each interview group will comprise four to six parents/caregivers. The first part will be a group think-aloud session during which caregivers voice their thoughts and experiences whilst using Point OutWords, facilitated by a team comprising the research assistants who have worked with caregivers and their children, an autism parent member of the research team who is not a participant in the study, and an expert on qualitative data collection. Think-aloud methodology can provide a valid source of data on participants’ thinking [53]. The second part will be a group discussion, without the research assistants present, addressing the software, families’ experiences using it, and how it fits into their daily family routine.

**Clinician interviews**

Semi-structured interviews will take place post-intervention with four referring clinicians. Questions will address their views on the intervention and on recommending it to parents.

#### Secondary outcomes

##### Child observations

**Verbal Motor Production Assessment for Children (VMPAC)**

The VMPAC [54] assesses neuromotor integrity of the speech system and is standardised for ages 3–12 years [54]. Items are categorised into five subdomains: global motor control, focal oromotor control, sequencing, connected speech and language, and speech characteristics. The VMPAC is sensitive to change following speech or motor intervention [55, 56], and subtests have strong test-retest reliability (correlation .88–.90), inter-rater reliability (correlation .93–.99), and high validity [54]. It will be administered at baseline and post-intervention, and mean score differences will be compared between the intervention and control groups.

##### Parent reports

**Vineland Adaptive Behavior Scales II (VABS-2)**

The VABS [57] is a widely-used parent interview measuring adaptive behaviour, recommended as a test-retest measure in autism treatment studies [58] and normed for all ages [59]. Items are categorised into four primary domains: communication, daily living skills, socialisation, and motor skills, with maladaptive behaviour (internalising and externalising) also examined. The VABS is administered at baseline and post-intervention; changes in mean scores across time between the intervention and control groups will be used to assess treatment efficacy. Similarly to our use of the MSEL, the baseline scores will characterise for whom the treatment is effective.

**Parenting Stress Index IV (PSI-4)**

The PSI [60] is a 120-item parent-report questionnaire assessing the extent of stress in the parent-child dyad, with foci on three core domains of stress: child characteristics, parent characteristics, and situational/demographic life stress. Mean differences in scores collected at baseline and post-intervention will be compared between the intervention and control groups.

**Family Quality of Life Survey (FQoL)**

The FQoL [61] is a 25-item parent-report questionnaire assessing perceived satisfaction in terms of quality of family life. Five domains are assessed: family interaction, parenting, emotional well-being, physical/material well-being, and disability-related support. Mean differences in scores collected at baseline and post-intervention will be compared between the intervention and control groups.

**Autism Impact Measure (AIM)**

The AIM [62] is a 25-item parent-report questionnaire that measures changes in core autism symptoms, and which has strong test-retest and cross-informant reliability [62]. Mean differences in scores collected at baseline and post-intervention will be compared between the intervention and control groups.

**Internal iPad data**

The iPad will record the child’s real-time finger movements when they are using Point OutWords, for the duration of the intervention period [30]. Changes in manual motor skills will be measured by the following parameters: (1) visuomotor targeting error is measured as the Euclidean (straight-line) distance between the initial point of contact with the screen and the nearest extent of the target puzzle piece, or the centre of the target key if in Type mode, (2) time of release and position of release relative to target are recorded, (3) motor efficiency is measured as variance of linear speed in the direction of motion, and variance of angular speed (the rate of change in direction of motion over time), (4) accuracy in movement will be measured as a ratio of the actual drag path to the shortest possible path length, and (5) the number of failed attempts to drag puzzle pieces and the overall time taken to successfully complete a piece are also recorded.

#### Other outcomes

##### Parental feedback, clinical and spoken language information

A structured parent-report questionnaire will be offered post-intervention, developed from the pilot study [30] to explore enjoyment of the app, engagement with other iPad or computer games, other therapies and to report languages used at home in addition to English (if any).

### Procedure

Participant eligibility will be assessed by the clinical lead, and staff under her direction. Research assistants will ascertain consent, allocate participants according to an independent randomisation procedure (https://SealedEnvelope.com/), and administer assessments, including recording a 12-min video of child and parent in free play for later scoring with the DCMA and BOSCC. The Principal Investigator (PI) received formal training in DMCA and BOSCC coding, which he passed on to the research assistants. The research assistants are trained to a high standard in psychometric and qualitative administration and coding, and receive regular supervision from the PI, the clinical lead, and qualitative specialist. Members of the research team responsible for data collection have completed CPFT Good Clinical Practice training. Parents will receive training to deliver the intervention and will be asked to keep a diary. Parents will be asked to complete the optional think-aloud session and focus group.

### Data management

Data will be anonymised and stored on a restricted shared drive in the CPFT network, to which only the research team will have access. Per the informed consent, anonymised data will be retained permanently (except in the case of withdrawal during the study) and may be released to other researchers. Identifying data (e.g. video, voice) will be deleted after scoring or transcription. Data entry will be completed and checked by research staff. Video data (for DCMA and BOSCC) and voice recordings (think-aloud, focus group, clinician interviews, and diaries) will be stored on a password-protected laptop computer at Nottingham Trent University. Physical data will be stored in locked cabinets within CPFT, accessible only by the research team. Internal data from Point OutWords will be pseudonymised and stored on a file server. The trial statistician and research team will undertake statistical analysis of the data. Only the clinical staff will have access to clinical records. As a small-scale feasibility study, this protocol does not include a data monitoring committee.

### Statistical analysis plan

Results will be analysed by the intention-to-treat principle. Guidelines and standard practices for each outcome measure will be used to impute values for missing items in individual datasets. For example, guidelines for the SRS-2 recommend using the median of the items answered. Any systematically missing subscales within feasibility candidate measures will inform the decision as to which measures would be carried forward to a full-scale RCT. This feasibility trial aims to determine the sample size for the full RCT. Test-retest variances in internal iPad data and external outcome measures will be entered into a standard calculation of statistical power to inform the sample size of a full RCT. Participant flow in terms of recruitment and retention, reported in line with the Consolidated Standards of Reporting Trials (CONSORT) statement for non-pharmacological interventions, will also inform the sample size for a large RCT. The trial statistician will perform the power and sample size calculations. Statistical analysis will be conducted in R.

We will explore the predictability of treatment effects based on the baseline measures. Findings may shed light on whether the intervention is more suited to certain profiles of autism than others. Inclusion criteria for a subsequent full trial would be fixed accordingly. Standardised scores from psychometric assessments will be analysed using mixed-model analysis of variance with time point (pre-intervention, post-intervention) as the within-subjects factor, and treatment (intervention, control) as the between-subjects factor. The internally logged outcome measures of visuomotor accuracy in Point OutWords will be analysed using three separate exploratory analyses of covariance, with Point OutWords usage time, number of distinct Point OutWords sessions, and real time as covariates. Participants’ own responses will be discriminated from caregivers’ model inputs by a statistical clustering method. Fidelity will be assayed using descriptive reports of content, and duration and number of intervention sessions completed.

#### Qualitative data and analysis

Data from parent diaries, focus groups and interviews will be transcribed and thematically analysed. A partially inductive and partially deductive (based on themes from our pilot study [30]) coding frame will be developed, using the analytical stages of Braun and Clarke [63]. Qualitative analysis will be evaluated using indicators of confidence and relevance [64] to ensure rigour, transparency, and accountability.

#### Video coding

Research assistants or others qualified for research scoring of the instruments in question will code the 12-min video recordings of parent and child play with the DCMA and the BOSCC coding frameworks. They will regularly co-rate the videos to maintain inter-rater reliability. To account for natural inter-rater differences, the same rater will code the pre and post scores, blind to the time point.

## Discussion

Point OutWords was developed in collaboration with children with autism and their therapists and caregivers [30], to include engaging jigsaws, high-contrast graphics, and exogenous cues to help capture and maintain attention, and customisable reinforcement prompts. The interface was adapted to accommodate open-loop visuomotor control (a visuomotor impairment common in people with autism), eliminating a major source of users’ frustration. Working from autistic cognitive strengths, Point OutWords aims to enhance motor skills and to promote representation of objects as sequences (of iconic puzzle pieces, symbolic letters, or symbolic sounds), prerequisite abilities that can support communicative development.

There is a need for therapies that address underserved subpopulations of autism and can be delivered with ease at a low cost. For children with autism who have motor impairment and greater receptive than expressive communication, our new intervention could fill this gap in services were it shown effective in a full-scale RCT. Before such a large-scale study can be framed, a feasibility trial is a necessary step to evaluate recruitment and retention rates and identify actions for reducing attrition. Feasibility trials are especially helpful if the proposed population, in this case, autism with motor impairment, has not previously been specifically tested [65]. Attrition poses a particular challenge with respect to the control group, but we hope that explaining the study’s contribution to science, regularly telephoning families to offer support, and offering the prospect of using Point OutWords after participation in the trial will motivate families to complete the study. As the acceptability of the control apps is a likely predictive factor in reducing attrition in the control group, with advice from parents of autistic children we have selected iPad apps that are enjoyable and fun for children and families. These control apps were selected based on the absence of Point OutWords skills and tasks, as the inadvertent involvement in similar therapy or training in a control group can confound results as recently reported in one large-scale trial [12].

Variance in outcome measures will be an outcome measure itself, used to inform on the suitability of certain psychometric assessments and the sample size in a full-scale RCT. The proportion of missing data in assessments will demonstrate which measures are acceptable to children and their caregivers. For example, in a previous study [19] children were not compliant with an oral motor assessment (Com DEALL Oro Motor Assessment [66]) possibly because of sensory sensitivities around the mouth. The last decade and a half of research on early intervention in autism has spurred the development of new measures sensitive to change from which the current trial benefits, such as the BOSCC [47] and the AIM [62]. Furthermore, we use a wide range of assessments to comprehensively ascertain the impact of the Point OutWords intervention, including measures of restricted and repetitive behaviour (BOSCC, SRS, AIM); some studies have found that improvement in communication skills can have a knock-on effect on the reduction of restricted/repetitive behaviours [9, 67]. Along the same lines, measuring an intervention’s outcomes, not just on autistic symptom severity but on individual and family well-being and daily functioning, has recently been underscored [68], so we include measures of parent stress (PSI), family quality of life (FQoL), and children’s challenging behaviour (maladaptive behaviour subdomain on VABS-2).

Qualitative data from diaries, interviews, and focus groups will help identify barriers to successful delivery of the intervention, allowing such issues to be resolved. The Point OutWords intervention is flexible therapy, not constrained to an environment or a time of day, which can be delivered when the child’s mood is most conducive to learning. Feedback about how Point OutWords slots into the daily family routine will be used to enhance the social validity of the intervention design.

As autism is a heterogeneous condition it is unlikely that one style of intervention will address all individuals, nor all aspects of its symptomatology within each individual. Our intervention for motor and communication skills facilitated by Point OutWords can complement other therapies that address communication and social skills more directly. Indeed, the answer will lie in cumulative effectiveness research and the use of common outcome measures across trials so that systematic reviews and meta-analyses can be easily conducted in the future [2]. The current trial prepares for this prospect, adopting measures such as the DCMA and BOSCC used in the PACT-G trial [69], and VABS-2 and the MSEL used in the Early Start Denver Model trial [12] and many other studies of autism. While the current trial is not sufficiently scoped to ascertain whether the Point OutWords intervention is effective in improving communication skills in children with autism, it can inform the design of larger RCTs, not only for Point OutWords but also for other early interventions in autism.

### Trial status

NIHR protocol number: 40703; Recruitment began on 12 March 2019 and is expected to be completed by December 2019.

### Monitoring

#### Trial Management Group and Patient Steering Committee

M.K. Belmonte (academic PI and overall project lead), E.J.L. Weisblatt (clinical PI), C. Días (parent co-investigator) and J. Foster (qualitative research specialist) will oversee the trial’s progress. The Trial Management Group will communicate amendments to testing or recruitment criteria to the rest of the research team. There are no interim analyses planned. In addition, we are setting up a Patient Steering Group comprising four parent representatives, which will convene quarterly. C. Días heads the Patient Steering Group and will report to the Trial Management Group.

#### Adverse Effects

Research staff will monitor participants for adverse effects, such as stress associated with the intervention, during fortnightly telephone calls with participants. One possible adverse effect might arise from overuse of the iPad, to a degree that could displace reciprocal social interaction between child and parent. To address this possibility, parents will be reminded that Point OutWords is a joint activity involving them and their child, not just the technology and their child. Safety reporting for adverse effects will be followed in line with CPFT Good Clinical Practice.

#### Auditing

Trial conduct will be audited monthly by the PI and the clinical lead. In accordance with Good Clinical Practice, a site folder will be held at the City Care Centre within the CPFT Integrated Children’s Service, containing copies of correspondence with families, the protocol, and a delegation log specifying the responsibilities of research team members. Case report forms for each participant will be used to record personal information, treatment allocation, dates of assessments and iPad drop-off, and relevant information collected during telephone calls during the intervention period e.g*.* if the child was feeling sick. Recruitment data are uploaded monthly to the NIHR Clinical Research Network Portfolio by a research assistant so that CPFT can provide additional support if the trial falls behind schedule.

#### Dissemination

We aim to publish methodological lessons and quantitative and qualitative trial results in peer-reviewed scientific journals. Preliminary results would be presented at the 2020 annual meeting of the International Society for Autism Research. Our web site [16] will be updated to inform parents and clinicians of the trial findings. Findings will be reported at CPFT multidisciplinary meetings and at a meeting for individuals with autism, their families, teachers, and therapists. Findings will be added to our trial registration at ISRCTN. Per standard NIHR contractual terms, the trial’s funder will have authority over publication, such authority not to be unreasonably applied. CPFT as the Sponsor will not have authority over publication. Authorship is available to members of the project team, and on request to others who have referred patients (e.g. clinicians) or collected or analysed data (e.g. students). Each empirical publication will include a statement of author contributions. There will be no use of professional writers.

## Trial Sponsor details

Cambridgeshire and Peterborough NHS Foundation Trust

Elizabeth House

Fulbourn Hospital

Cambridge

United Kingdom

CB21 5EF

Telephone: +44 1223 219400

Email: foi@cpft.nhs.uk

## Supplementary information

**Additional file 1.** SPIRIT 2013 Checklist: Recommended items to address in a clinical trial protocol and related documents

## Data Availability

Data sharing is not applicable to this article as no datasets were generated or analysed for this protocol study. Participant-level data will be available at a later date. Although no specific reuse of participants’ data in future studies is contemplated a priori*,* this possibility is allowed by the consent document except of course in instances where the parent has withdrawn their child’s data from the study: “By agreeing to participate in this study, you are giving us permission to release anonymous, non-identifying data to other researchers for their use in legitimate scientific research. These data may be posted on the World Wide Web for other scientists to download. At any time whilst the study remains ongoing – even after your child’s participation in the study may have ended – you may withdraw your consent, and in this case we would delete your data from the study dataset and from the set of anonymised data that we release in future.” No biological specimens will be collected for any outcomes.

## Abbreviations

ABA: Applied behaviour analysis
ADOS-G: Autism Diagnostic Observation Schedule - Generic
AIM: Autism Impact Measure
BOSCC: Brief Observation of Social Communication Change
BPVS-3: British Picture Vocabulary Scale III
Com DEALL: Communication developmental eclectic approach to language learning
CONSORT: Consolidated standards of reporting trials
CPFT: Cambridgeshire and Peterborough Foundation Trust
FQoL: Family Quality of Life Survey
MSEL: Mullen Scales of Early Learning
NHS: UK National Health Service
NICE: National Institute for Health and Care Excellence
NIHR: National Institute of Health Research
PSI-4: Parenting Stress Index IV
RCT: Randomised controlled trial
SRS-2: Social Responsiveness Scale II
VABS-2: Vineland Adaptive Behaviour Scales II
VMPAC: Verbal Motor Production Assessment for Children
